# PET clinical study of novel antipsychotic LB-102 demonstrates unexpectedly prolonged dopamine receptor target engagement

**DOI:** 10.1038/s41386-024-01951-x

**Published:** 2024-10-16

**Authors:** Dean F. Wong, Ganesh B. Chand, Nicole Caito, Anna Eramo, Vincent T. Grattan, Mark S. Hixon, Ginger Nicol, Erin Lessie, Zachary Prensky, Hiroto Kuwabara, Lucy Tian, Ines Valenta, Thomas H. Schindler, Gerhard Gründer, Andrew R. Vaino

**Affiliations:** 1 Mallinckrodt Institute of Radiology, and Department of Radiology, Washington University in Saint Louis, St. Louis, MO USA; 2 Department of Psychiatry, Washington University in Saint Louis, St. Louis, MO USA; 3 Department of Neurology, Washington University in Saint Louis, St. Louis, MO USA; 4 Department of Neuroscience, Washington University in Saint Louis, St. Louis, MO USA; 5 LB Pharmaceuticals Inc., New York, NY USA; 6 Mark S. Hixon Consulting LLC, San Diego, CA USA; 7 Johns Hopkins University Department of Radiology, Baltimore, MD USA; 8 Central Institute of Mental Health, Department of Molecular Neuroimaging, Medical Faculty Mannheim, University of Heidelberg, Mannheim, Germany

**Keywords:** Target validation, Schizophrenia

## Abstract

Regulation of dopamine activity has important clinical consequences, most notably in schizophrenia. LB-102, *N-*methyl amisulpride, is a novel dopamine D_2/3_/5-HT_7_ inhibitor being developed as a treatment for schizophrenia and other psychiatric disorders. The characteristic that is common to all current antipsychotics is their engagement of D_2_ dopamine receptors. The goal of this study was to measure the dopamine receptor occupancy of orally administered LB-102 at three different doses (50, 75, and 100 mg as single doses and 50 and 100 mg as multiple doses) and at different timepoints in healthy volunteers using positron emission tomography (PET) with ^11^C raclopride as a radiotracer. Results of this study (NCT04588129) showed that steady-state once daily oral dosing of 50 mg LB-102 afforded striatal dopamine occupancy (RO) in the desired 60–80% range consistently over the course of 24 h. Contrary to the often observed relationship between RO vs plasma concentrations, maximum dopamine RO significantly lagged maximum plasma concentration and showed little variability under steady state conditions. A similar phenomenon has recently been reported with a non-racemic version of amisulpride [1]. LB-102 was generally safe and well-tolerated at all doses. Results of this study were used to inform dosing in a subsequent Phase 2 clinical study in schizophrenia patients.

## Introduction

It has been theorized that the pharmacologic mechanism of action for antipsychotic drugs is (at least some) antagonism of dopamine receptors in the brain [2–4]. For first and most second generation antipsychotics dopamine receptor occupancy (RO) in the 60–80% range is typically desired to maximize improvements in symptoms of schizophrenia [5], with the risk of extrapyramidal symptoms (EPS) —which range from acute involuntary movements or dystonia to more chronic and debilitating symptoms like akathisia and tardive dyskinesia—accelerating as dopamine RO surpasses 80% [6].

Amisulpride is a dopamine D_2_ (K_i_ 2.8 nM) and D_3_ (K_i_ 3.2 nM) receptor antagonist that also inhibits 5-HT_7_ receptors (22 nM K_i_), which has been implicated in the treatment of mood disorders [7–9]. Amisulpride is used as a treatment for depression SEP-4199 is a non-racemic, 85:15 *R*:*S* version of amisulpride that has been evaluated in a Phase 2 clinical study in bipolar disorder [10–12] Because of amisulpride’s poor membrane permeability, high doses are required to achieve plasma concentrations associated with dopamine RO > 60%. For example, 630 mg amisulpride resulted in just over 70% dopamine RO (plasma amisulpride concentration not reported) in patients with schizophrenia [13]. In vitro studies of LB-102 have demonstrated its interaction with 5-HT_7_ receptors [14].

**Fig. 1:**
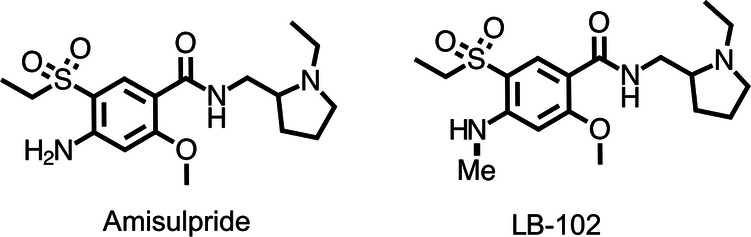
Chemical structures of amisulpride and LB-102.

LB-102 (Fig. 1) is a *N* methylated version of amisulpride, a dopamine inhibitor developed in the 1980s and approved in more than 50 countries outside the United States for the treatment of schizophrenia. Clinically, amisulpride is one of the most effective drugs to treat schizophrenia. In a recent meta-analysis of 32 drugs in 54,000 participants [15], amisulpride was second only to clozapine in antipsychotic efficacy and was the second-best tolerated drug as measured by time to all cause discontinuations [16].

In in vitro studies LB-102 and amisulpride had similar CNS receptor binding profiles and bound to dopamine and 5-HT_7_ receptors equivalently [13]. In animal behavioral assays of schizophrenia, LB-102 was equivalent to, or better than, amisulpride and it displayed a similar pharmacokinetic profile when dosed orally [17]. In a phase 1 clinical study (NCT04187560) LB-102 was safe and generally well-tolerated up to 150 mg/day [18].

The present work describes a positron emission tomography (PET) study of orally dosed LB-102 with ^11^C raclopride. The goal of this study was to measure dopamine RO in the brains of healthy volunteers to inform dosing in a planned Phase 2 clinical study of LB-102 in patients with schizophrenia. The relationship between RO and plasma LB-102 concentration was measured, as were safety and tolerability of LB-102 after single and multiple dose(s). Based on modeling work previously presented [13] and on preclinical animal models we did expect a dissociation between plasma concentration of LB-102 and dopamine RO but did not know the magnitude of this in humans, which was the purpose of this study. Data from studies of dopamine RO in healthy volunteers have demonstrated fidelity to data obtained in schizophrenia patients [19, 20].

## Participants and methods

This study was conducted at the Mallinckrodt Institute of Radiology (MIR) PET facility at Washington University School of Medicine (WUSM) in St. Louis during 2020 and was approved by the WUSM Institutional Review Board in accordance with ethical standards established in the 1964 Declaration of Helsinki. Figure S1 depicts a schematic of the study design.

Upon providing informed consent, participants were screened to confirm eligibility within 14 days of baseline PET (inclusion/exclusion criteria in Supplement Information). Safety monitoring included vital signs (blood pressure and heart rate, oral body temperature, and respiratory rate), 12-lead electrocardiograms (ECGs), clinical laboratory testing (hematology, clinical chemistry, and urinalysis), adverse event (AE) assessments, and physical examination. The Columbia-Suicide Severity Rating Scale (C-SSRS) was used to assess suicide risk. The overall schedule of assessments for the study are outlined in Table S1.

### Enrollment

Twenty-four volunteers were screened and sixteen were enrolled based on inclusion/exclusion criteria. All enrolled participants completed the study and were included in the analyses. Sixteen healthy volunteers (7 female, 9 male) ages 18 to 55 years and with a body mass index (BMI) between 18 and 30 kg/m^2^ were enrolled in the study. No enrolled subjects dropped out of the study. Sizing of this study was based on prior experience with PET imaging studies and did not take statistical powering assumptions into account. Table 1 summarizes demographic data of study participants.

**Table 1:** Summary of demographic information of study participants.

	50 mg 1X	75 mg 1X	100 mg 1X	50 mg 4X	100 mg 4X
n	4	4	4	2	2
Age (yrs)	31 (4)	37 (10)	28 (8)	23 (1)	45 (11)
Weight (kg)	68 (7)	73 (15)	82 (7)	82 (8)	85 (3)
BMI (kg/m^2^)	23 (1)	26 (3)	26 (3)	27 (4)	27
Male/female	3/1	3/1	1/3	2/0	2/0
White	4	3	2	1	
African American	1	2			
Asian				1	
White/Asian					2

Values for age, BMI, and weight are averages, standard deviation in parentheses.

### Study design

This was an open label study, adaptive design, study. Cohort 1 was chosen to be a 50 mg single oral dose based on prior preclinical and human PK non-PET imaging studies [13]. The 75 mg and 100 mg doses were determined empirically after observation of prior doses and were determined empirically with the goal of bracketing 60–80% dopamine RO. Timing of PET scans, starting at 2.5, 7.5, and 23.5 h were selected for the 50 and 100 mg single doses and multiple doses to capture dopamine RO for a full 24 h period after dosing. In the 75 mg single dose arm the 7.5 h scan was replaced by a scan starting at 47.5 h to better understand the decay kinetics of dopamine RO resulting from LB-102. This additional time point did not affect interpretation of the results beyond reassuring that dopamine RO would not persist indefinitely.

On providing informed consent, subjects were screened to determine study eligibility within 14 days of baseline PET scan. Within two weeks after baseline PET, subjects checked in to the WashU Center for Translational Research Unit (CTRU) to receive their first dose of LB-102. This inpatient stay allowed for frequent medical monitoring after dosing and after PET scans.

Subjects in Cohorts 1 to 3 received single doses and subjects in Cohort 4 received once daily doses over 4 days—a Phase 1 clinical study of LB-102 [14] demonstrated that LB-102 plasma concentration reached steady state the morning of dosing day 4. The 4-day dosing was designed to capture receptor occupancy under steady state conditions.

**Fig. 2:**
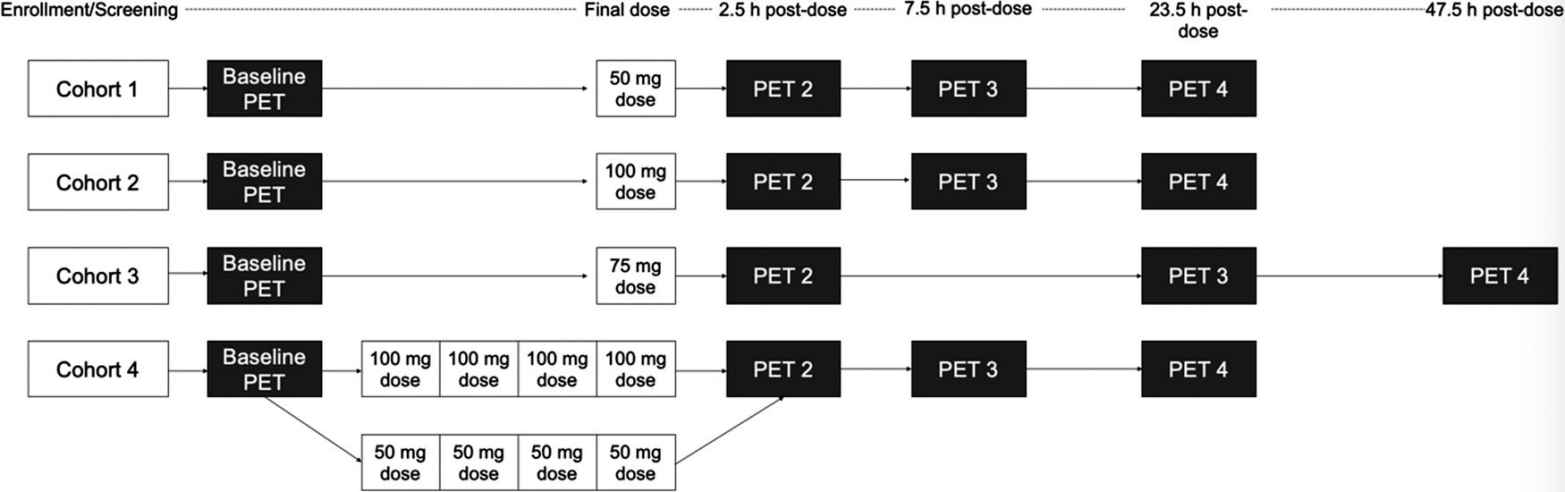
Schematic of study design.

In Cohorts 1-3, a single dose of LB-102 was administered orally as a powder in capsule on day 1 of the study and in Cohort 4 once a day for four days. Dose administration was scheduled for 8:00 AM (±1 h) each dosing day, following the collection of vital signs and a 12-lead ECG. The dose was given with water and subjects had the option to eat breakfast after dosing. All cohorts underwent four PET scans, including one baseline scan prior to administration of LB-102 followed by three post-dose scans at varying timepoints. In cohorts 1 and 2, post-dose scans started at 2.5 h, 7.5 h, and 23.5 h after dosing. For cohort 3, post-dose scans started at 2.5 h, 23.5 h, and 47.5 h after dosing. Subjects in Cohort 4 underwent post-dose scans following the final dose (day 4) of LB-102 at 2.5 h, 7.5 h, and 23.5 h after final dosing. PET scans were acquired over 90 min and values reported are a time-weighted average of values obtained. Subjects were discharged from the CTRU following their final PET scan. A schematic of the trial design is presented in Fig. 2.

In Cohort 1, all four subjects were dosed with 50 mg LB-102. In Cohort 2, all subjects were dosed with 100 mg LB-102, and in Cohort 3 all subjects were dosed with 75 mg LB-102. Initially, all four subjects in Cohort 4 were to receive 100 mg LB-102; the first two subjects demonstrated higher than expected dopamine receptor occupancy at 100 mg QD for four days, approaching 90%. It was decided to reduce dosing to 50 mg for the final two subjects out of an abundance of caution.

### Radiotracer and positron emission tomography

Subjects received a total of 4 PET scans with ^11^C raclopride as a tracer. The mean injected activity was 14.1 ± 0.31 mCi (SEM). One baseline PET scan (pre-dose) and 3 PET scans were obtained at several time points following final LB-102 dose. Each PET scan lasted 90 min and collected 30 frames (four 15 s, four 30 s, three 1 min, two 2 min, five 4 min, and twelve 5 min). PET scans were obtained on a PET-CT Siemens Vision and MRI on a 3 T Prisma. A T1-weighted 3D MPRAGE MRI sequence was acquired on each subject using the following parameters: repetition time, 2400 ms; echo time, 2.62 ms; flip angle, 8; slice thickness, 1 mm; field of view, 256 mm × 256 mm; number of axial slices, 176.

### Image processing and derivation of PET outcome

PET images were analyzed using PMOD [21]. T1-weighted MRI was used for co-registration of PET images. Images were normalized into standard space. Volumes of interest (VOIs) from Hammers template [22] (caudate, putamen, thalamus, temporal lobe, and cerebellum) were applied to PET frames to obtain regional time-activity curves (TACs). TACs were used for tracer kinetic modeling and binding potential non-displaceable (BP_ND_) was computed at caudate, putamen, thalamus, and temporal lobe taking cerebellum as a reference region. In PMOD, kinetic modeling analyses were performed using the simplified reference tissue model (SRTM) [23] and Logan [24] graphical reference tissue model.

RO was obtained for each of the 3 PET scans (scans 2, 3, or 4) following the baseline PET scan as follows:

$${RO}=\,100\,X\frac{{{BP}}_{{ND}}{{Baseline\; scan}}_{i}{{Region}}_{j}-{{BP}}_{{ND}}{{postdose\; scan}}_{i}{{Region}}_{j}}{{{BP}}_{{ND}}{{Baseline\; scan}}_{i}{{Region}}_{j}} \%$$

Where the i = 2^nd^, 3^rd^, or 4^th^ PET scan and region j is the caudate, putamen or thalamus region.

To assess the reproducibility of results from above two methods, the RTGA 10T60, RTGA 10T90 [25], and MTRM2 [26] were further used using IDEA [27]. In IDAE, VOIs were determined using Freesurfer [28]. Cerebellum VOIs were manually edited to exclude non-cerebellar elements include local sinus.

^11^C raclopride was produced at the MIR cyclotron facility at high specific activity (940.41 ± 35.40 Ci/mmol) and average injected mass (5.42 ± 0.14 µg). Subjects all had head fixation with an individualized thermoplastic mask. All PET scans were carried out after a short bolus injection of the radiotracer followed by dynamic PET imaging of 30 frames over 90 min on a Siemens PET CT Vision where the attenuation scan was carried out first by a short non contrast CT scan. Reconstruction employed an iterative 3D time of flight (True X + TOF (Ultra HD) 8 iterations, 5 subsets, Allpass Filter) and dynamic scan durations were four 15 s, four 30 s, three 60 s, two 120 s, five 240 s, twelve 300 s starting with the IV injection of the radiotracer.

Each subject received a baseline PET in the absence of LB-102, then repeat PET scans according to the Study Design described above with up to three post single dose (cohorts 1 -3) and after four days of chronic dosing as inpatients in the WUSTL CTRU (Center for Translational Research Unit).

### Safety

Safety was monitored during the study using the following procedures and assessments at regular intervals: blood pressure, heart rate, oral body temperature, 12-lead ECGs, clinical labs (hematology, clinical chemistry, and urinalysis), adverse event (AE) assessments, and physical examinations. Subjects were given the Columbia-Suicide Severity Rating Scale (C-SSRS) to assess suicide risk. Follow-up by telephone was conducted on Day 3 for subjects receiving single doses and on Day 7 for subjects receiving multiple doses.

### Data analysis

LB-102 RO was determined as the amount of ^11^C raclopride displaced by LB-102 using PET at baseline (pre-dose Day 1) and starting at 2.5 h, 7.5 h, and 23.5 h for Cohorts 1 and 2, at baseline and starting at 2.5 h, 23.5 h, and 47.5 h for Cohort 3, and at baseline and at 74.5 h, 79.5 h, and 95.5 h for Cohort 4 in the caudate, putamen, thalamus, and temporal cortex.

## Results

**Fig. 3:**
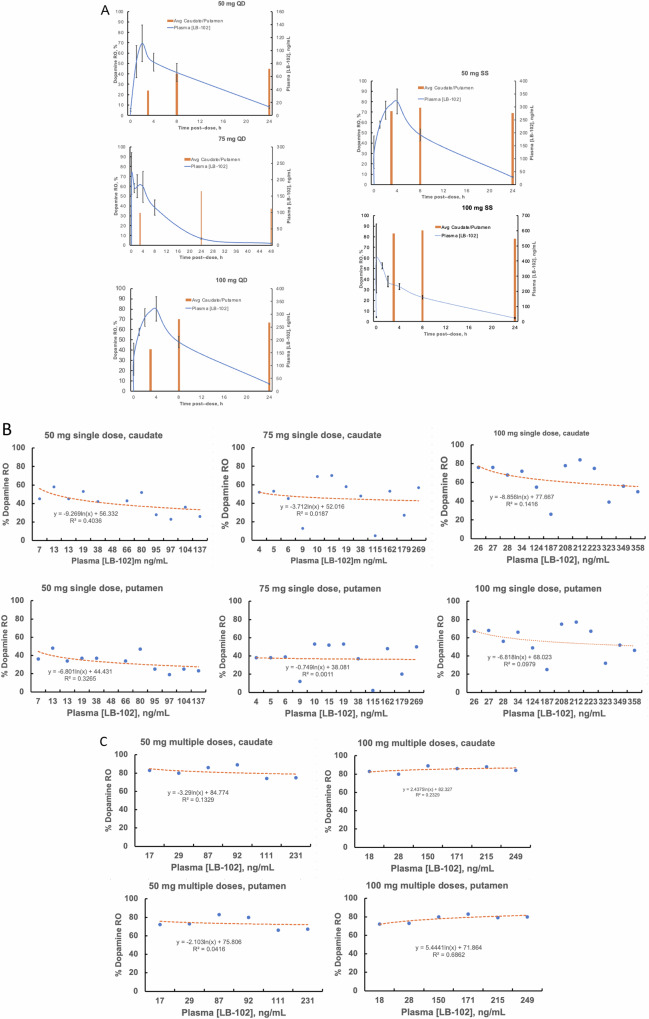
Dopamine receptor occupancy versus plasma LB-102 concentration. **A** Receptor Occupancy and Plasma concentration of LB-102 (0-24 hr. post drug) striatal dopamine %RO (average of caudate and putamen, calculated using the SRTM method) and plasma PK data for subjects (*n* = 4/group ± SEM) dosed with single doses of LB-102 (QD) or four QD doses of LB-102 (SS). Dopamine Receptor Occupancy, in both caudate and putamen, plotted versus observed plasma concentration at time of scan for (**B**) single doses LB-102 and (**C**) multiple doses LB-102.

Receptor occupancy in four regions of the brain (caudate, putamen, thalamus, and temporal cortex) was measured starting at 2.5, 7.5, and 23.5 h post-dose (for 50 mg and 100 mg doses) and starting at 2.5, 23.5, and 47.5 h post dose for (75 mg doses). A total of 64 PET scans were acquired, 4 per subject. Plasma concentrations were contemporaneously measured at regular intervals after dosing. Results of the RO and PK data after either a single dose LB-102 or 4 daily doses of LB-102 (steady state conditions) are depicted in Fig. 3A. Average subject activity ranged from 789 to 1158 Ci/mmol and injected masses ranged from 3.9 to 6.2 µg; there were no difference between subjects by ANOVA, thus differences in measured occupancy could not have been due to the mass of the injected raclopride, nor to relevant differences in precision of the injected radioactivity both within and across subjects and cohorts. Table S3 shows SRTM dopamine %RO at each time point for each subject in each region.

Figure 3A shows the Receptor Occupancy, calculated using the SRTM method vs. plasma concentration of LB-102 (0–24 h. post drug) The striatal dopamine %RO was the average of caudate and putamen and plasma PK data for subjects (*n* = 4/group ± SEM) dosed with single doses of LB-102 (QD) or four QD doses of LB-102 (SS).

Calculated SEM values for RO were < 3%, markedly lower than the SEMs observed for PK measurements for which they approached 20%. Figure 3B and C show percent dopamine receptor occupancy versus plasma LB-102 concentration for each dose together with a best logarithmic fit showing poor correlation.

Receptor occupancy is a first-order process that ought to be defined by a monotonically increasing logarithmic function of drug concentration, for examples as noted with brexpiprazole [29].

Average RO, measured using the SRTM method, for each subject in each region are presented in Table S2. Note that RO data were calculated using five different methods: SRTM, Logan, RTGA 10T60, RTGA 10T90, and MRTM2. There were no meaningful statistical differences between the respective methods and data obtained using the SRTM are presented as representative.

Treatment-related adverse events reported by participants in this study were mild or moderate in severity, similar to previous reports. No episodes of acute dystonia were reported or observed, consistent with a prior study in healthy adults in which acute EPS was observed at higher doses of LB-102 (75 mg twice daily i.e. 150 mg/day). As in prior study, LB-102 was generally safe and well-tolerated, and clinical lab values were unremarkable at all doses, save for prolactin, which was transiently elevated in all participants. Prolactin was measured at baseline and at last visit, average prolactin at baseline was 7.5 ng/mL (SD 2.9) which increased, on average, to 30.8 (SD 13.0) at final visit. In this, and a prior study of LB-102 [3], there were no clinically observable effects of prolactin elevation (for example: galactorrhea or menstrual irregularities). There were no abnormal ECG readings; the average change in QT_cf_ from baseline was -1 ms. No changes in blood cell counts or tests of liver or kidney function nor suicidal ideation were observed. Participants were not permitted to smoke, consume alcohol, cannabis, or illicit substances, so pharmacologic interactions with these substances were not evaluated. A summary of adverse events is given in Table 2.

**Table 2:** Summary of treatment emergent adverse effects.

Adverse event	Occurrence	Relation to treatment
Headache	3	Possibly
Dizziness	1	Possibly
Restlessness	2	Unlikley
Anxiety	1	Unlikley
Acid Reflux	1	Not Related
Nasal Congestion	1	Not Related
Sore Throat	1	Not Related

## Discussion

Of note in Fig. 3 is that for LB-102 dopamine RO is remarkably disconnected from plasma concentration: that is, dopamine RO remains significant after plasma concentrations have dropped below 10 ng/mL. This disconnect is consistent with previously presented data on the relationship between LB-102 plasma concentration and dopamine mediated efficacy of LB-102 in animal models of SCZ [30], though the observation of 40% dopamine RO a full two days after a single 75 mg dose of LB-102 was unexpected. Results from the study did suggest that once-a-day dosing of LB-102 could be effective in treating schizophrenia (amisulpride is typically dosed twice daily).

Results of this PET study showed that under steady-state conditions 50 mg LB-102 administered orally once a day provides approximately the same dopamine receptor occupancy —~70%—as reported in the literature for 300–400 mg amisulpride [20]. Importantly, this 70% RO is in the midpoint of the 60–80% range typically sought for dopamine inhibitors in the treatment of schizophrenia and shows very little variability (~10%) over the course of 24 h. Consistent dopamine receptor engagement may be one of the reasons schizophrenia treatment with long acting injectable therapeutics, which provide consistent exposure of drug, are typically superior to daily dosing (which results in daily peak-trough variability) [31].

The unusual disconnect between RO and PK during the study departs from many typical receptor occupancy studies seen with both first- and second-generation antipsychotics; for example as noted with brexpiprazole [31], lumateperone [21], and risperidone [32] in which plasma concentration of drug more closely tracks brain dopamine RO than do the examples in Fig. 3A–C. A recent study by Hopkins et al. [1]. on a non-racemic version of amisulpride (SEP-4199^1^) reported a similar dislocation between plasma drug concentration and dopamine RO though, as in the present work, a mechanistic explanation is unclear. As Fig. 3 shows, the relationship between plasma concentration and dopamine receptor occupancy is not linear and higher plasma concentrations of LB-102 are not always associated with higher receptor occupancy, for example, up to 80% dopamine RO with a plasma LB-102 concentration of 10 ng/mL (from a C_max_ of 400 ng/mL). Even with the 75 mg dose there was persistent activity at 48 h post single dose as well as similar or increased occupancy at 50 and 100 mg single doses. This may be due to the increased lipophilicity of LB-102 and increased residence time in brain, which may be due to more than just lipophilicity alone (the measured Log P for amisulpride was 1.52 while that for LB-102 was 1.72; the measured pKas for amisulpride and LB-102 were 9.32 and 9.36, respectively), though to what extent lipophilicity governs equilibration to the brain for LB-102 is unclear. This asymmetry between brain and periphery is in-line the idea of the “Inside-out neuropharmacology” observed in particular with nicotinic drugs [33], which seeks to explain the lag between initial dosing of psychiatric drugs in plasma and receptor engagement in a pharmacokinetically separate compartment; that is LB-102 CNS concentration lags plasma concentration creating a temporal dislocation between plasma concentration and drug efficacy. Prior work on modeling amisulpride/LB-102 based on animal models suggested that the rate of influx for amisulpride/LB-102 was greater than the rate of efflux which could be responsible for the observed prolonged receptor occupancy observed. The role of the methyl group in this equilibrium is unclear and was beyond the scope of the current work. Additionally, it is possible that this prolonged receptor occupancy is due to a metabolite of LB-102, though data to date indicate that LB-102 is not appreciably (<5%) metabolized. The metabolism of LB-102 will be studied more completely in a subsequent study. For LB-102 this may be an advantage, not a confound, as consistent RO has been demonstrated to improve outcomes in treating schizophrenia.

**Strengths** of this work include a balanced and remarkably similar ^11^C raclopride injected activity and injected mass. Single oral doses, based on prior experience and analogy to amisulpride, had RO of 60–80% at ~400 mg and the chronic dosing of LB-102 replicated this at only 50 mg. Based on clinical observations in this study LB-102 was not associated with EPS or QT interval alterations and the drug was generally well-tolerated; both of these important markers of safety will be more closely observed in subsequent clinical studies.

**Limitations** of this study include a small number of RO observations at steady state but allowed bracketing 50 and 100 mg. The measure of the residence time of LB-102 is indirectly inferred from observation of D_2_/D_3_ RO. Future work may include more direct measurements of LB-102 in brain to further validate the hypothesis of its potential improved brain kinetics and potential to be an effective antipsychotic with antidepressant action while enjoying a low side effect potential.

**In summary**, LB-102 demonstrated prolonged brain dopamine RO consistent with known dopamine antagonists that successfully treat schizophrenia. This study was conceived both as proof of concept that LB-102 would be an effective dopamine inhibitor in humans and also to inform dosing in a Phase 2 clinical study of LB-102 in schizophrenia patients. Based on the present work, doses in a 350 patient clinical study (NCT06179108) of 50 mg, 75 mg, and 100 mg QD LB-102 were selected.

## Supplementary information

Supplemental Material

Table S1

## Footnotes

1 SEP-4199 was under development for bipolar depression; a Phase 3 clinical study (NCT05227209) was terminated in December 2023.
