# Supplementary material for: PET clinical study of novel antipsychotic LB-102 demonstrates unexpectedly prolonged dopamine receptor target engagement
Source: Neuropsychopharmacology. 2024 Oct 16;50(2):372–7. doi: 10.1038/s41386-024-01951-x (PMC11632090; doi:10.1038/s41386-024-01951-x)
Supplement: Supplementary file 1 — Supplemental Material [file 41386_2024_1951_MOESM1_ESM.pdf]

PET clinical study of novel antipsychotic LB-102 demonstrates unexpectedly prolonged dopamine receptor engagement

Supplementary Information

Table of Contents

TABLE S1: CONSORT FLOW DIAGRAM 3
TABLE S2: LB-102-002 INCLUSION AND EXCLUSION CRITERIA 4
FIGURE S1: SCHEMATIC OF STUDY DESIGN 7
TABLE S3: SCHEDULE OF STUDY ASSESSMENTS 8
TABLE S4: STRM DOPAMINE %RO AT EACH TIME POINT FOR EACH SUBJECT IN EACH REGION 9
TABLE S5: SPECIFIC ACTIVITIES AND WEIGHT ^{11}C RACLOPRIDE INJECTED, COHORT AVERAGES 10
TABLE S6: SPECIFIC ACTIVITIES AND WEIGHT ^{11}C RACLOPRIDE INJECTED, INDIVIDUAL VALUES 10

Table S1: Consort Flow Diagram

CONSORT 2010 Flow Diagram

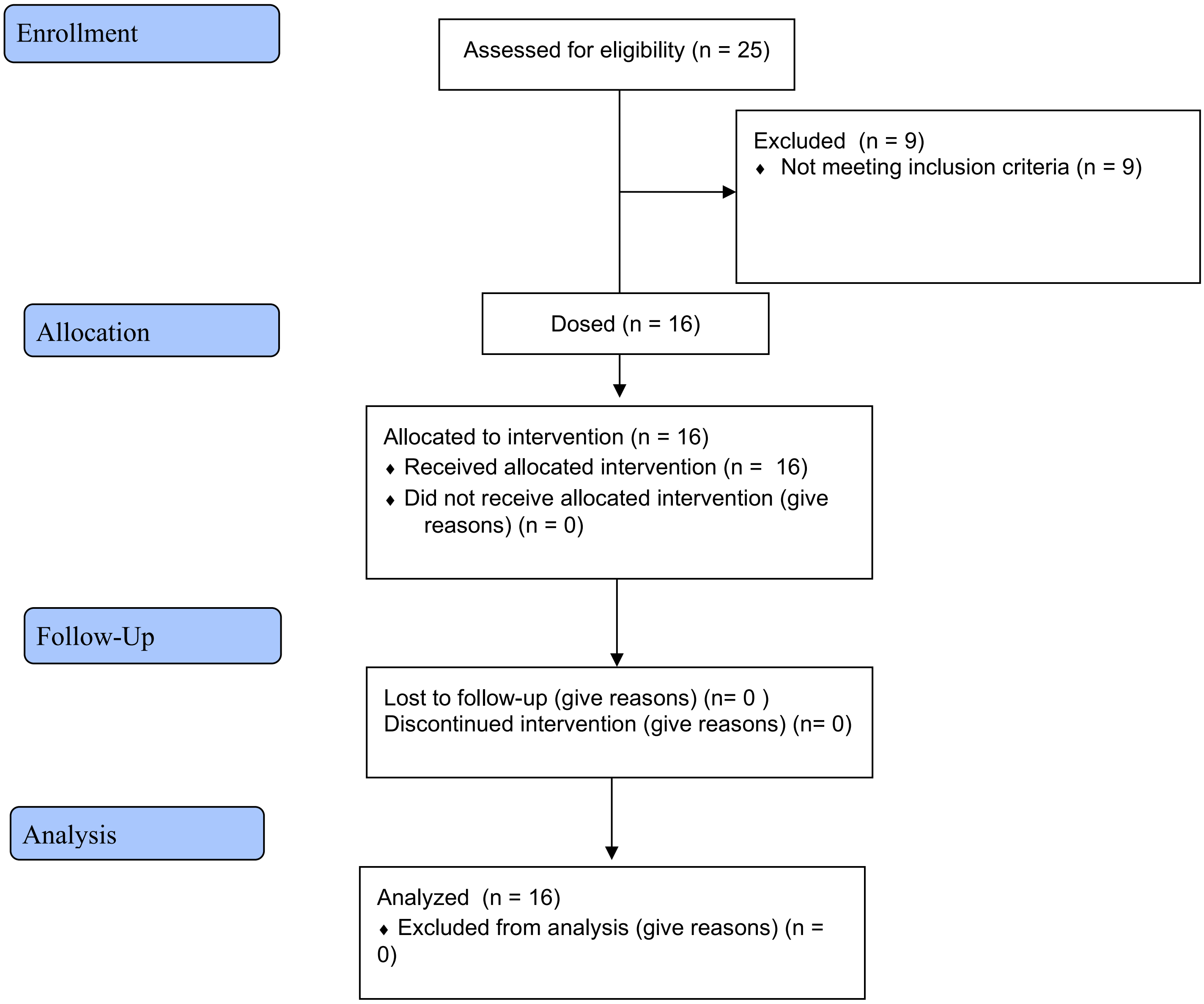

Table S2: LB-102-002 inclusion and exclusion criteria

Main Criteria for Eligibility:

Inclusion Criteria:

A subject will be eligible for inclusion in the study if he or she meets the following criteria:

1. Competent to provide informed consent.
2. Voluntarily provide informed consent and Health Insurance Portability and Accounting Act (HIPAA) Authorization in accordance with local regulations and governing Institutional Review Board (IRB) requirements prior to any procedures or evaluations performed specifically for the sole purpose of the study.
3. Healthy adult female and male subjects between 18 to 55 years of age inclusive at the screening visit.
4. Body Mass Index (BMI) ≥ 18 and ≤ 30 kg/m^2 at screening visit.
5. Subjects must be in good general health as determined by medical history and physical examination with no clinically significant medical findings and no history of significant medical disease (e.g., cardiovascular, pulmonary, renal, etc.) or acute condition with the past 30 days, as determined by the study investigators.
6. Have normal clinical laboratory test results and ECG, which are not considered to be clinically significant by the Investigator.
7. Females participating in the study:
    a. Either must be of non-childbearing potential [surgically sterilized: hysterectomy, bilateral tubal ligation, salpingectomy, and/or bilateral oophorectomy at least 26 weeks before the Screening Visit] or postmenopausal. Menopause is defined as being amenorrhoeic for at least 2 years with plasma follicle-stimulating hormone (FSH) levels in the post-menopausal range at screening, based on the central laboratory's rang**es; OR**
    b. **Females of child-bearing potential mus**t have a negative pregnancy test and be not breastfeeding at Screening and use either abstinence or an accepted contraception method during the treatment period and for an additional period of 30 days after the end of investigational treatment. For this study, accepted contraception methods include:
        i. condom plus spermicide
        ii. condom plus diaphragm
        iii. condom plus cervical cap or female condom

    iv. hormonal contraceptives
    v. intrauterine device
    vi. partner vasectomy and a use of barrier contraception methods

8. If male, subject must be surgically sterile or practicing at least 1 of the following methods of contraception, from initial study drug administration through 90 days after administration of the last dose of study drug:
    a. Have had a vasectomy (at least 6 months earlier);
    b. Use of a double-barrier contraception method, defined as male use of a condom and female use of a barrier method (e.g., contraceptive sponge, spermicidal jelly or cream with or without a diaphragm);
    c. Partner use of hormonal contraceptives (oral, parenteral, vaginal, or transdermal) for at least 3 months prior to study drug administration;
    d. Partner use of an intrauterine device;
    e. Complete abstinence from sexual intercourse;
    f. Male subjects not practicing complete abstinence must use a condom as a required form of birth control (in addition to at least 1 of the other methods listed above, if desired) in order to protect partners of male participants from exposure to study drug.
9. If male, subject must agree to abstain from sperm donation through 90 days after administration of the last dose of investigational drug.

Exclusion Criteria:

A subject will be excluded from the study if he or she meets the following criteria:

1. Are pregnant or lactating.
2. Have a history or presence of significant cardiovascular, respiratory, hepatic, renal, gastrointestinal, endocrine, neurological or psychological/psychiatric disorders which, in the opinion of the Investigator, increases the risk of the study drug or may confound the interpretation of study measures.
3. Clinically significant abnormal findings on physical examination or vital signs as determined by PI.
4. Individuals with pacemakers, aneurysm clips, shrapnel, or other restricted implanted metallic devices will be excluded from study. All subjects complete the standard MRI screening questionnaire prior to MRI.
5. History or presence of psychiatric or neurological disease or condition as determined by the PI.
6. History of seizures.
7. Subject with any history or current evidence of suicidal behavior.

8. Unwilling to complete any planned study assessments.
9. Have a history of blood donation in excess of 500 mL of blood within 30 days prior to Screening.
10. Have received treatment with an investigational drug or device within 30 days prior to Screening.
11. Have a positive test for Human Immunodeficiency Virus (HIV) antibodies 1 and 2, Hepatitis B Surface Antigen (HBsAg) or Hepatitis C Virus (HCV) antibody.
12. Any subject who is known to be allergic to the study drug or any components of the study drug.
13. The subject has a fasting blood glucose ≥ 126 mg/dL or hemoglobin A1c (HbA1c) ≥ 6.5% at Screening.
14. The subject has a history of QT prolongation or dysrhythmia or a family history of prolonged QT interval or sudden death.
15. Clinically significant abnormal finding on ECG and/or evidence of any of the following cardiac conduction abnormalities at Screening:
    a. Heart rate < 40 bpm and > 100 bpm (based on the ECG reading)
    b. QTcF interval > 450 msec for males and females
    c. PR interval ≥ 200 msec
    d. Intraventricular conduction delay with QRS duration > 120 msec
    e. Evidence of second- or third-degree atrioventricular block (AVB)
    f. Electrocardiographic evidence of complete left bundle branch block (LBBB), complete right bundle branch block (RBBB), or incomplete LBBB
16. Any subject who has a positive Urine Drug Screen test on the Day 0 or Day 1 Visit, unless in the Investigator's (Principal Investigator or Sub-Investigator) documented opinion, the positive test does not signal a clinical condition that would impact the safety of the subject or interpretation of the trial results. The Investigator (Principal Investigator or Sub-Investigator) must provide their documented opinion to the Sponsor's Medical Monitor, and then the Sponsor (CMO) within 24 hours of their decision.
17. Any subject who has an Alcohol Breathalyzer test result deemed positive by the Investigator (Principal Investigator or Sub-Investigator) on the Day 0 or Day 1 Visit, unless in the Investigator's (Principal Investigator or Sub-Investigator) documented opinion, the positive test does not signal a clinical condition that would impact the safety of the subject or interpretation of the trial results. The Investigator Principal Investigator or Sub-Investigator) must provide their documented opinion to the Sponsor's Medical Monitor, and then the Sponsor (CMO) within 24 hours of their decision.

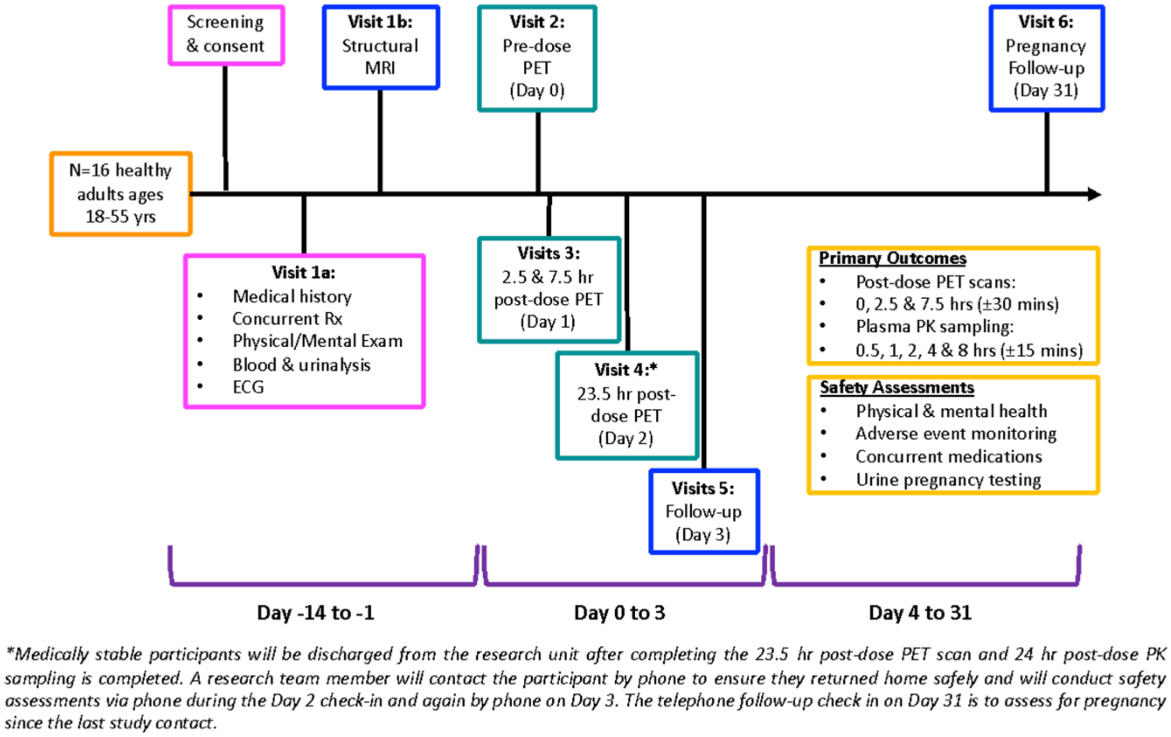

Figure S1: Schematic of study design

Table S3: Schedule of Study Assessments

Study Visit	1	2	3	4	5	6
**Study Day(s)**	**-14 to -1**	**0**	**1**	**2**	**3**	**31**
**Location**	**OP**	**OP**	**IP**	**IP/OP**	**OP**	**OP**
Informed Consent	X					
Medical History	X	X				
Demographics	X					
Height, Weight, BMI	X					
Physical Exam[1] & Vital Signs[2]	X[1]	X[1]	X[2]	X[2]		
Structural MRI[3]	X					
Screening labs*	X	X		X*		
Urine Drug Screen[@]	X	X	X			
Alcohol Breathalyzer		X	X			
Serum HbA1c	X					
Serum Prolactin	X			X		
HIV, HBsAg, and HCV	X					
C-SSRS	X			X		
12-Lead ECG[4]	X	X		X		
Pregnancy Test[5]	X	X	X	X		
Plasma PK sampling[6]			X	X		
Dose LB-102[7]			X			
Concurrent Meds[8]	X	X	X	X	X	
Adverse Events[8]		X	X	X	X	
PET Scan[9]		X	X	X		
Phone check-in[10]				X	X	X

BMI=Body Mass Index; CSSRS=Columbia Suicide Severity Rating Scale; ECG=Electrocardiogram; FSH=Follicle-Stimulating

[1] Physical examination can be performed at screening or on Day 0.
[2] Vital Signs are measured at Screening, Day 0 (at time of PET scan, ±30 min), Day 1 (pre-dose), and at the time of each PET scan (±30 min, Visits 3 and 4).
[3] Screening MRI can be done any time before Day 0. An MRI obtained in the prior 6 months for research purposes can substitute for this screening scan.
[4] ECG is done at Screening, Day 0 (prior to PET scan, ±30 min), and Day 2 (24 h post-dose, ±30 min).
[5] Serum pregnancy test at Screening and Urine pregnancy test on Days 0-2 for all females of childbearing potential.
[6] Plasma PK samples are collected on Day 1 (pre-dose), and at the following times post dose (±15 min): 0.5, 1, 2, 4, 8, and 24 h (Day 2).
[7] Participants are required to fast for approximately 12 hours prior to Day 1 dosing.
[8] Concomitant Medication and Adverse Event Assessment will be recorded once per day on the days indicated.
[9] For Cohorts 1-2, PET scans are completed at 2.5, 7.5, and 23.5 (±30 min) hours post oral dose of LB-102.
[10] Day 2 follow-up call is done in the evening when the subject returns home. Day 3 follow-up call is done in the morning.
[*] On Day 2, blood is collected for laboratory tests (hematology, clinical chemistry, urinalysis).
[@] Urine Drug Screens will occur at Screening and Days 0-1.
[+] Or Serum FSH for post-menopausal females.

Table S4: STRM dopamine %RO at each time point for each subject in each region

		3 h				8 h				24 h				48 h			
		**S1**	**S2**	**S3**	**S4**	**S1**	**S2**	**S3**	**S4**	**S1**	**S2**	**S3**	**S4**	**S1**	**S2**	**S3**	**S4**
50 mg	Caudate	26	28		23	52	42	36	43	58	45	53	45				
	Putamen	23	25		19	47	37	25	34	48	36	37	34				
	Thalamus	40	42		35	48	31	42	39	39	23	45	28				
	Temporal lobe	20	16		26	32	14	20	28	30	11	19	18				
		**S1**	**S2**	**S3**	**S4**	**S1**	**S2**	**S3**	**S4**	**S1**	**S2**	**S3**	**S4**	**S1**	**S2**	**S3**	**S4**
100 mg	Caudate	39	56	50	26	75	78	84	55	76	72	76	68				
	Putamen	32	52	46	25	67	75	77	49	68	66	67	56				
	Thalamus	28	42	34	31	40	49	46	49	26	43	39	39				
	Temporal lobe	18	17	20	10	24	17	20	15	13	23	22	15				
		**S1**	**S2**	**S3**	**S4**	**S1**	**S2**	**S3**	**S4**	**S1**	**S2**	**S3**	**S4**	**S1**	**S2**	**S3**	**S4**
75 mg	Caudate	27	53	57	5					70	58	69	48	53	45	52	13
	Putamen	20	48	50	2					52	53	53	37	38	39	38	12
	Thalamus	31	44	37	5					64	37	36	21	16	25	20	0
	Temporal lobe	13	21	28	0					45	17	20	27	4	16	16	0
		**S1**	**S2**			**S1**	**S2**			**S1**	**S2**						
100 mg SS	Caudate	84	88			86	89			80	83						
	Putamen	80	79			83	80			73	72						
	Thalamus	50	78			51	80			48	69						
	Temporal lobe	19	32			18	25			22	31						
		**S1**	**S2**			**S1**	**S2**			**S1**	**S2**						
50 mg SS	Caudate	75	74			78	76			75	73						
	Putamen	67	66			69	70			66	63						
	Thalamus	44	37			40	43			38	56						
	Temporal lobe	18	26			17	25			35	34						

Table S5: Specific activities and weight ^{11}C raclopride injected, cohort averages

Cohort Average				
**Cohort**	**Specific Activity (Ci/mmol)**	**SEM**	**Injected Mass (ug)**	**SEM**
1	927.52	62.83	5.15	0.39
2	934.11	96.82	5.50	0.31
3	879.36	59.29	5.78	0.17
4	1019.85	58.07	5.23	0.25

Table S6: Specific activities and weight ^{11}C raclopride injected, individual values

Subject Average				
**Participant**	**Specific Activity (Ci/mmol)**	**SEM**	**Injected Mass (ug)**	**SEM**
01S0001	811.86	147.83	6.03	1.19
01S0002	864.71	147.50	5.41	0.26
01S0003	988.54	131.09	3.86	0.81
01S0005	1060.21	65.64	4.99	0.37
01S0006	970.26	97.07	5.91	0.83
01S0009	944.11	169.06	5.28	0.54
01S0011	789.12	143.60	5.66	0.27
01S0012	1032.93	343.70	5.13	0.84
01S0013	974.23	213.07	5.25	0.36
01S0014	977.91	23.62	5.68	0.16
01S0016	788.00	51.71	6.04	0.07
01S0017	777.32	95.89	6.15	0.52
01S0018	1001.77	78.86	5.69	0.30
01S0020	970.12	86.27	5.21	0.33
01S0021	949.55	119.79	5.37	0.50
01S0024	1157.96	174.61	4.67	0.75
